# COUPLING AUDITORY CUES AND BILATERAL TRANSAURICULAR VAGUS NERVE STIMULATION IN PARKINSON’S DISEASE WITH FREEZING OF GAIT: AN OPEN-LABEL FEASIBILITY STUDY

**DOI:** 10.2340/jrm.v58.45165

**Published:** 2026-03-04

**Authors:** Andrea DI MAIO, Mario MELONI, Jae-Jung SONG, Vincenzo DI LAZZARO, Massimo MARANO

**Affiliations:** 1 Unit of Neurology, Neurophysiology, Neurobiology and Psychiatry, Department of Medicine, University Campus Bio-Medico of Rome, Rome; 2 Fondazione Policlinico Universitario Campus Bio-Medico, Rome; 3 Neurology Unit, Azienda Ospedaliera Universitaria di Cagliari, Cagliari, Italy; 4 Department of Otorhinolaryngology-Head and Neck Surgery, Korea University Guro Hospital, Seoul; 5 Institute for Health Care Convergence Center, Korea University Guro Hospital, Seoul; 6 Neurive Institute, Neurive Co Ltd, Seoul, Korea

Parkinson’s disease (PD) is the fastest-growing neurological disorder worldwide. Freezing of gait (FOG) – an abrupt inability to initiate the step – represents one of its most severe complications (1, 2). Physical therapy is the gold standard (1, 3). The use of auditory cueing (AC) is generally effective on FOG, although a subset of patients shows limited or inconsistent responsiveness (3, 4). Among non-invasive brain stimulation strategies, vagus nerve stimulation (VNS) has emerged as a possible strategy to treat gait troubles and FOG (5). Indeed, various small studies have investigated its efficacy in PD (5–9). Through the stimulation of the auricular branch of the vagus nerve, transauricular VNS (taVNS) activates monoaminergic, cholinergic, and GABAergic ascending systems (5). Given the growing evidence on the use of taVNS in PD and in rehabilitation, the ACOUSTIC-PD (Auditory cueing COUpled with vagus STImulation for Conditioning gait parameters in PD) pilot study was designed to explore the feasibility, tolerability, and preliminary efficacy of combining taVNS with AC in a well-characterized group of individuals with PD and a history of FOG.

## MATERIALS

Twenty-one patients were consecutively enrolled in the study if affected by idiopathic PD according to the International PD and Movement Disorders Society criteria, with a documented history of FOG, with an age between 50 and 75, able to walk unaided (Hoehn and Yahr scale ≤ 3), and without clinical evidence of cognitive impairment. Patients with hearing impairment, comorbidities potentially affecting gait performance (e.g., musculoskeletal, vestibular, or peripheral neuropathic disorders), and with any other active or past neurological issue involving sensory-motor and cognitive domains were excluded.

**Fig. 1:**
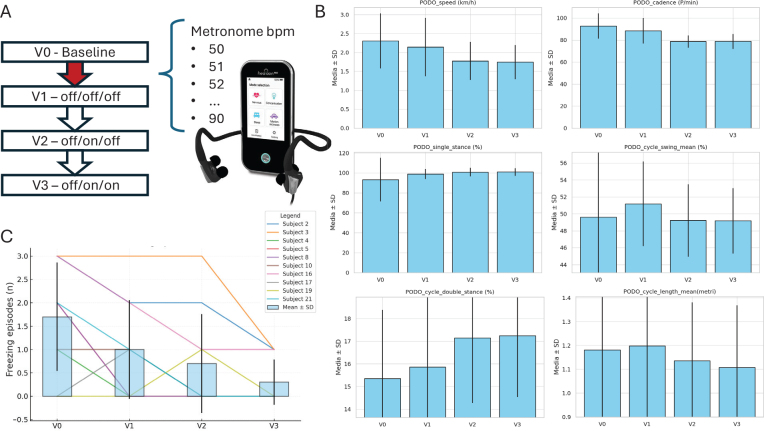
(A) study design, V0 (baseline), V1 (off-medication, off-AC, off-taVNS), V2 (off-medication, on-AC, off-taVNS), V3 (off-medication, on-AC, on-taVNS), red arrow (AC conditioning phase); (B) gait analysis data from PODOsmart system across visits; (C) FOG episodes across visits.

Bilateral taVNS was delivered at internal tragus and cymba conchae through the Healaon pro device (Neurive, Seoul, Korea; http://eng.neurive.com/). Patients received painless bilateral taVNS at an individual intensity (i.e., based on the sensory threshold, eliciting a non-painful tingling sensation) and at frequency and pulse width of 30 Hz and 1000 µs (6). AC was delivered as metronome sounds through Healaon earplugs. Each patient identified their own beat-per-minute (bpm) value during the training phase of the experiment and both taVNS intensity and AC bpm values were kept constant across visits (Fig. 1).

Subjects underwent 4 consecutive visits in fixed order, performed after an overnight withdrawal of dopaminergic therapies (medication-off state). The baseline assessment (V0) included the Movement Disorder Society Unified PD Rating Scale (MDS-UPDRS) part 3, the Montreal Cognitive Assessment (MoCA), and the new FOG questionnaire (nFOG-Q) (10). Patients also were tested with 5-m Timed Up and Go (TUG) tests per visit, executed in triplicate (i.e., the mean of the 3 trials was used for statistical analyses). TUG trials were then repeated at V1 (following AC conditioning), V2 (during AC stimulation) and V3 (during AC plus taVNS) (see Fig. 1).

The AC conditioning included 15-min simple gait training with AC on an individual bpm basis, when the patient became confident with the device and AC along the walking path, supported by therapists if necessary.

Gait parameters were automatically recorded through the PODOSmart system (i.e., smart insoles), collecting data on speed (km/h), gait cycle phases (%), swing length (m), and cadence (step/min). The difference between the selected bpm and the mean cadence (Delta [bpm – cadence]) was also collected. All TUGs were videorecorded, and FOG episodes were manually identified through a blinded visual inspection performed by 2 independent raters. Adverse events were also recorded.

Given the exploratory nature of the study, a control/sham condition was not included and sample size calculation was not required. Repeated measures of non-parametric data (Shapiro–Wilk) across the 4 visits (V0–V3) were compared using the Friedman test, with post-hoc pairwise comparisons (Wilcoxon signed-rank) and correction for multiple testing (Bonferroni). Kendall’s W effect size (V0–V3) was also documented. Statistical analyses of freezing episodes were restricted to individuals reporting FOG episodes during the experiment. Data are reported as mean ± standard deviation. Significance was set at alpha ≤ 0.05 (two-tailed). This study was approved by the local Ethics Committee.

## RESULTS

Enrolled patients (*n* = 21) had a mean age of 65.95 ± 7.96; 33% (*n* = 7) were women. The group presented a moderate disease stage overall (disease duration of 7.80 ± 5.20 years, MDS-UPDRS part 3 of 36.9 ± 16.8, Hoehn and Yahr of 2.35 ± 0.46), a LEDD of 703.5 ± 386, and a MoCA of 26.2 ± 3. The selected metronome bpm was 72.3 ± 4.4. Although all 21 patients reported a history of FOG (mean nFOG-Q 10.5 ± 2), only 10 subjects reported FOG episodes during the experiment.

Statistical analysis demonstrated a significant main effect of the experimental conditions on gait parameters and on the number of freezing episodes. Speed (χ² = 32.5, *p* < 0.001), cadence (χ² = 25.7, *p* < 0.001) swing (χ² = 18.8, *p* < 0.001), step length (χ² = 9.09, *p* = 0.028), and delta (bpm-cadence) (χ² = 21.63, *p* < 0.001) decreased, while double stance (χ² = 20.26, *p* < 0.001) and single stance (χ² = 22.18, *p* < 0.001) increased at V0 vs V1, V1 vs V2, V0 vs V2. The mean number of FOG episodes also showed significant changes across visits (χ² = 14.83, *p* = 0.002), with a significant reduction at the combined AC + taVNS visit (V3: 0.3 ± 0.48) vs baseline (V0: 1.7 ± 1.6) *(p* = 0.050) (Fig. 1, Table I and Table SI). Gait symmetry and mean clearance did not change significantly. No adverse events were recorded.

**Table I:** Gait parameters observed during the study

	V0	V1	V2	V3	Friedman *p*-value	V0 vs V3	
						Bonferroni *p*-value	Kendall’s W
Gait video inspection (*n*=10)*							
Total FOG, *n*	17	10	7	3	0.002	0.051	0.450
Mean FOG	1.7 ± 1.6	1 ± 1.05	0.7 ± 0.6	0.3 ± 0.48			
Gait analysis (*n* = 21)							
Symmetry (%)	91.15 ± 1.32	92.20 ± 1.58	91.95 ± 1.21	92.90 ± 1.16	0.446	0.825	0.042
Speed (km/h)	2.26 ± 0.16	2.07 ± 0.15	1.74 ± 0.10	1.72 ± 0.09	< 0.001	0.0015	0.505
Cadence (p/min)	92.57 ± 2.71	87.45 ± 2.49	78.25 ± 1.08	78.50 ± 1.28	< 0.001	0.0017	0.367
Double stance (%)	15.36 ± 0.62	15.95 ± 0.68	17.15 ± 0.56	17.20 ± 0.53	< 0.001	0.0022	0.355
Single stance (%)	65.38 ± 0.41	65.96 ± 0.00	67.13 ± 0.21	67.16 ± 0.08	< 0.001	0.0022	0.224
Swing (%)	34.61 ± 0.41	34.03 ± 0.00	32.86 ± 0.21	32.81 ± 0.02	< 0.001	0.0022	0.224
Length (m)	0.80 ± 0.02	0.78 ± 0.04	0.74 ± 0.03	0.72 ± 0.03c	0.028	0.168	0.202
Delta (bpm–cadence)	–15.60 ± 22.40	–15.10 ± 10.42	–5.90 ± 6.10	–6.15 ± 6.34	< 0.001	0.0015	0.322

* *n* = 10, FOG-positive while testing subjects; visits sharing the same letter are not significantly different according to pairwise Wilcoxon signed-rank tests with Bonferroni correction (alpha=0.05). MDS UPDRS: Movement Disorders Society Unified Parkinson’s Disease Rating Scale 1; FOG: freezing of gait (episodes).

## DISCUSSION

The combined intervention of AC and taVNS in PD was well tolerated, with no adverse event reported. The task constraints (i.e., external rhythmic pacing) reduced gait speed and cadence, and increased stance – possibly improving gait stability. Because clinically meaningful endpoints such as falls, patient-reported mobility, or real-world walking performance were not assessed, no conclusions can be drawn regarding functional benefit beyond laboratory-based gait organization. The marked reduction in delta (bpm–cadence) indicated a closer synchronization between targeted (bpm) and actual cadence (step per minute), reflecting improved rhythmic-motor coupling. The addition of taVNS did not significantly modify the effects of AC on gait parameters. However, our results confirm the efficacy of AC on FOG and offer preliminary support for the hypothesis that combining these techniques may confer additional benefits, as suggested by early efficacy signals. One possible explanation, not testable with the current design, is that AC is responsible for motor timing and rhythmic entrainment (top-down influence) (11, 12), while taVNS may act through a bottom-up effect (13). Interestingly, PD with FOG display diminished cortical delta activity (14), and bilateral taVNS has been shown to enhance frontal delta oscillations in healthy individuals (15).

Limitations of this study include the open-label design, small sample size, and absence of a control arm. Moreover, observed changes could reflect the effect of practice, fatigue, habituation, or expectation due to the fixed visit order, and of the short-distance laboratory task. The sequential, non-randomized design precluded formal testing of efficacy differences or additive effects beyond AC, while the off-medication condition limits generatability. As such, these findings should be considered exploratory. The experimental condition, constraining patients on a rhythmic cadence, prevented any other possible conclusion on the independent effect of taVNS on gait – which was not tested.

In conclusion, while AC is a well-established technique for improving gait and reducing FOG in PD, the combined intervention was feasible and safe and showed preliminary baseline-referenced signals that warrant further controlled investigations.

## Supplementary Material



## Trial

IRB registration number 142.24 CET2 cbm.
