# Supplementary material for: COUPLING AUDITORY CUES AND BILATERAL TRANSAURICULAR VAGUS NERVE STIMULATION IN PARKINSON’S DISEASE WITH FREEZING OF GAIT: AN OPEN-LABEL FEASIBILITY STUDY
Source: J Rehabil Med. 2026 Mar 4;58:45165. doi: 10.2340/jrm.v58.45165 (PMC12969777; doi:10.2340/jrm.v58.45165)
Supplement: Supplementary file 1 [file JRM-58-45165-s1.pdf]

Supplementary material has been published as submitted. It has not been copyedited, or typeset by Journal of Rehabilitation Medcine

Table SI. Post-hoc pairwise comparisons (Wilcoxon signed-rank tests, Bonferroni-corrected)

**Outcome**	**V0 vs V1**	**V0 vs V2**	**V0 vs V3**	**V1 vs V2**	**V1 vs V3**	**V2 vs V3**
**Speed (km/h)**	0.084	*0.0015*	*0.0015*	*0.0022*	*0.0022*	0.764
**Cadence (P/min)**	0.091	*0.0017*	*0.0017*	*0.0022*	*0.0022*	0.892
**Double stance (%)**	0.113	*0.0022*	*0.0022*	*0.0022*	*0.0022*	0.815
**Single stance (%)**	0.128	*0.0022*	*0.0022*	*0.0022*	*0.0022*	0.834
**Swing (%)**	0.128	*0.0022*	*0.0022*	*0.0022*	*0.0022*	0.834
**Step length (m)**	0.342	0.061	0.168	0.094	0.121	0.763
**Delta [bpm–cadence]**	0.402	*0.0015*	*0.0015*	*0.0022*	*0.0022*	0.886
